# Diffusion kurtosis imaging of brain white matter alteration in patients with coronary artery disease based on the TBSS method

**DOI:** 10.3389/fnagi.2024.1301826

**Published:** 2024-02-15

**Authors:** Tong Li, Rui Qin, Cuicui Li, Lin Li, Ximing Wang, Li Wang

**Affiliations:** ^1^Department of Radiology, Shandong Provincial Hospital Affiliated to Shandong First Medical University, Jinan, China; ^2^Department of Health Management Center, Shandong Provincial Hospital Affiliated to Shandong First Medical University, Jinan, China

**Keywords:** coronary artery disease, cognitive impairment, MRI, diffusion kurtosis imaging, tract-based spatial statist

## Abstract

**Object:**

The aim of our study was to examine the alterations in microstructure in patients with coronary artery disease (CAD) and cognitive impairment (CI) using diffusion kurtosis imaging (DKI). Additionally, we aimed to investigate the potential correlation between DKI parameters and cognitive function.

**Materials and methods:**

A total of 28 CAD patients and 30 healthy controls (HC) were prospectively enrolled in our study. All participants underwent routine and diffusion sequences of head imaging. DKE software was utilized to generate various diffusion kurtosis imaging parameters (DKI), including kurtosis fractional anisotropy (KFA), mean kurtosis (MK), axial kurtosis (AK), radial kurtosis (RK), fractional anisotropy (FA), and mean diffusivity (MD). Nonparametric tests were conducted using tract-based spatial statistics (TBSS) to compare the parameter values between the two groups. The parameter values of the significantly different fiber tracts were extracted and correlated with the Mini-Mental State Examination (MMSE) and Montreal Cognitive Assessment (MoCA) scores.

**Results:**

Compared to the HC group, patients with coronary artery disease exhibited significant reductions in FA values in the bilateral Superior corona radiata, bilateral Anterior corona radiata, bilateral Posterior corona radiata, corpus callosum, left Posterior thalamic radiation, right Posterior limb of internal capsule, Anterior limb of internal capsule, and Cerebral peduncle, as well as in the left Superior longitudinal fasciculus. Additionally, KFA values decreased in the bilateral Anterior corona radiata, bilateral Anterior limb of internal capsule, and Genu of the corpus callosum. The MK values decreased in the right Posterior corona radiata, Retrolenticular part of the internal capsule, Posterior thalamic radiation (including optic radiation), Superior longitudinal fasciculus, and left Posterior thalamic radiation (including optic radiation). Moreover, the RK values decreased in the bilateral Retrolenticular part of the internal capsule, right Posterior thalamic radiation (including optic radiation), and Superior longitudinal fasciculus, as well as in the left Superior longitudinal fasciculus and Posterior thalamic radiation (including optic radiation) (*p* < 0.01, TFCE corrected), while no significant differences were observed in other parameter values (*p* > 0.01, TFCE corrected). The FA values of the right posterior limb of the internal capsule (*r* = 0.610, *p* = 0.001) and the right cerebral peduncle (*r* = 0.622, *p* < 0.001) were positively correlated with MMSE scores. Additionally, a significant correlation between kurtosis and diffusion coefficient parameters (FA and KFA) was observed.

**Conclusion:**

CAD patients showed radial shrinkage and complexity of brain white matter microstructure. Whole-brain white matter analysis based on TBSS DKI can objectively reflect the characteristics of white matter damage in CAD patients, providing a basis for the auxiliary diagnosis of CAD with CI.

## 1 Introduction

Coronary artery disease (CAD) is a common cardiovascular condition characterized by atherosclerosis in the coronary arteries, leading to narrowing or blockage of blood vessels and resulting in myocardial ischemia, hypoxia, or necrosis. Clinical manifestations often include chest discomfort, chest pain, and fatigue. Coronary artery stenosis or occlusion can be identified through CT angiography (CTA) and coronary angiography. Neuroimaging typically reveals the formation of multiple ischemic lesions in the brain (Bretzner et al., 2021). CAD is associated with an increased risk of CI and progression to dementia (Mayda et al., 2011; Kovacic et al., 2012; Muqtadar et al., 2012). However, there is limited research on CI caused by CAD, and further discussion is needed regarding its neuroimaging changes. MRI plays a vital role as a noninvasive imaging tool in examining the damage to cerebral white matter (WM) networks in patients with CAD. This examination is crucial because WM damage serves as a predictor not only for cognitive decline in the elderly but also for future occurrences of ischemic stroke and myocardial infarction (MI) in CAD patients. Therefore, investigating the extent of WM damage in this population is of utmost importance for their overall health and well-being (Gerdes et al., 2006; Mayda et al., 2011; Defrancesco et al., 2013). Previous research has established a correlation between CAD, its associated cardiovascular risk factors, and damage to cerebral WM. This WM damage has been found to be independently associated with a higher risk of cognitive decline and progression to dementia. These findings emphasize the importance of addressing CAD and its risk factors in order to mitigate the potential negative impact on cognitive function and the development of dementia. By effectively managing CAD and its related factors, it is possible to reduce the risk of WM damage and its associated cognitive complications (Breteler et al., 1994; de Leeuw et al., 2002; Kral et al., 2013; O’Brien, 2014). In a study conducted by Zheng et al., it was found that macrostructural neuroimaging indices, such as WMH, were insufficient in fully explaining the cognitive impact of CAD (Zheng et al., 2012). Compared to macrostructural neuroimaging, diffusion kurtosis imaging parameters (DKI) is a highly sensitive technique that allows for more accurate observation of subtle changes in brain white matter microstructure. Compared to Diffusion Tensor Imaging (DTI), DKI, based on a non-Gaussian diffusion model, provides a more accurate fit to water molecule diffusion and is more sensitive to diffusion heterogeneity (Tan et al., 2019). Research has indicated that DKI is more promising than DTI in assessing the integrity of neural tissue in brain white matter regions with complex fiber arrangements. This is because DKI is closely associated with cellular microstructure, providing a more comprehensive understanding (Follin et al., 2019). TBSS, by extracting the fiber skeleton, enables the study of changes in brain white matter while avoiding the subjective and poorly reproducible aspects of ROI-based analysis of DKI data. This approach is more advantageous for studying white matter fibers (Smith et al., 2006). Many studies have previously explored the roles of different fiber bundles in brain function. For example, research has found that the corpus callosum facilitates information transmission and coordinates communication between the two hemispheres of the brain, promoting the development and maintenance of cognitive abilities (Douaud et al., 2007; Salo et al., 2009). The cingulum, mainly responsible for connecting cortical and subcortical regions, plays an important role in cognitive functions related to memory and language processing. Projection fibers, as well as the superior longitudinal fasciculus, also play crucial roles in the normal functioning of cognitive abilities in the brain (Pettersson et al., 2005). Our study hypothesizes that certain white matter fiber bundles, such as the corpus callosum and cingulum, may be damaged in CAD patients, thus mediating the occurrence of cognitive impairments.

We employed the TBSS method to analyze the characteristic changes in DKI parameters in CAD patients’ brain white matter and their relationship with cognitive function. Our study found significant differences in fractional anisotropy (FA) values, kurtosis fractional anisotropy (KFA) values, mean kurtosis (MK) values, and radial kurtosis (RK) values in certain brain regions between the two groups. For example, in the bilateral anterior corona radiata, bilateral anterior limb of the internal capsule, and genu of the corpus callosum, the KFA values were decreased in the CAD group compared to the HC group. This suggests that there are some alterations in the white matter fibers of CAD patients compared to the HC group, which may mediate the occurrence of cognitive dysfunction. The aim of our study was to explore the potential neuroimaging changes in CAD patients with cognitive dysfunction based on DKI sequences.

## 2 Method

A total of 28 patients with clinically diagnosed coronary artery disease were prospectively recruited from Shandong First Medical University Affiliated Provincial Hospital between December 2022 and June 2023. During the same period, 30 age-, gender-, and education level-matched individuals were recruited as a healthy control (HC) group. All participants underwent cranial MRI examinations, and a comprehensive assessment of cognitive function was conducted using the Mini-Mental State Examination (MMSE) and the Montreal Cognitive Assessment (MoCA) scale. The study enrolled patients with coronary artery disease (CAD) who met the following inclusion criteria: aged 35–75 years, clinically diagnosed with CAD confirmed by imaging examination, and able to undergo magnetic resonance imaging (MRI) scans. The exclusion criteria included patients with cerebral infarction, carotid artery stenosis or occlusion, contraindications for MRI, and consciousness or mental disorders. The control group consisted of volunteers without neurological or cardiovascular diseases who had undergone comprehensive cardiovascular examinations at our hospital. All participants underwent cardiac coronary computed tomography angiography (CTA) to assess their artery condition. Additionally, their cardiovascular risk factors, medical history, medication use, and thorough cardiovascular examination by a cardiologist were evaluated. This study received approval from the Ethics Committee of Shandong Provincial Hospital, affiliated with Shandong First Medical University. Prior to their participation, all participants provided written informed consent.

### 2.1 Neuropsychological examinations

The participants in the study were evaluated for their cognitive status using the Montreal Cognitive Assessment (MoCA) and Mini-Mental State Examination (MMSE) scales (Zhou et al., 2008). The MoCA scale assesses various cognitive functions, including visuospatial/executive abilities, naming skills, attention span, language proficiency, abstraction capabilities, memory recall, and orientation. These assessments were conducted following standardized procedures in a quiet environment. The maximum score for both scales is 30 points. Scores below 26 on the MoCA and below 24 on the MMSE indicate poor cognitive function. If the total score of MMSE and MoCA is less than 30 and the years of education are less than or equal to 12, then add 1 point to the total score.

### 2.2 Magnetic resonance imaging protocol

Whole-brain images were obtained at the Shandong Provincial Hospital Affiliated to Shandong First Medical University using a Siemens 3.0 T Prisma MR system and a 64-channel head coil for brain scanning. Participants were carefully positioned inside the machine, and foam padding was used to minimize any movement during the scanning process.

T1-weighted whole-brain magnetization-prepared rapid gradient echo (MPRAGE) images were collected to capture anatomical details using the following parameters: TR = 2,530 ms, TE = 2.98 ms, TI = 1,100 ms, FOV = 256 × 256 mm2, in-plane resolution = 256 × 256 mm2, flip angle = 7°, and 192 axial slices. Diffusion imaging was performed using spin–echo echo-planar imaging (SE-EPI). The parameters were set as follows: TR = 5,000 ms, TE = 95 ms, FOV = 224 mm × 224 mm, matrix size 110 × 110, slice thickness 2.0 mm, and 74 slices acquired simultaneously using the simultaneous multislice (SMS) technique with an acceleration factor of 2. Three *b*-values were used: *b* = 0, 2000, and 3,000 s/mm^2^, with 64 diffusion gradient directions.

### 2.3 Data preprocessing

We applied dcm2niigui to convert all diffusion data from DICOM format to NIFTI format, and Oxford University’s FSL software was used for motion and eddy current correction. We assessed the quality of the data and excluded datasets with severe image deformation, artifacts, and excessive noise. To compute DKI metrics, including KFA,MK, axial kurtosis (AK), and RK, as well as diffusion tensor metrics, including FA, mean diffusion (MD), and radial diffusion (RD), we used the DKE software.^1^ The TBSS analysis was performed on all parameter maps using the FSL-provided FMRIB58_FA template. All subjects’ images were registered and normalized to the Montreal Neurological Institute (MNI) standard space using nonlinear registration. The normalized images were averaged and skeletonized in MNI space, with a threshold of 0.2. This threshold was used to exclude gray matter or cerebrospinal fluid components and ensure the generation of a reliable white matter skeleton. Finally, the skeletonized maps of all subjects were obtained. Based on the parameter maps standardized to MNI space, the DPABI software package in MATLAB 2016b was utilized to extract the relevant parameter values of the brain regions where there are differences in fiber bundle parameters between two groups using Johns Hopkins University (JHU)-ICBM labels as a template.

### 2.4 Statistical analysis

We performed all data analyses using SPSS 22.0 statistical software. Normally distributed continuous variables are presented as the mean ± standard deviation (SD). The age, MMSE, and MoCA scores of the CAD and control groups were compared using independent samples *t-*tests. Nonparametric tests were used for nonnormally distributed data, and the chi-square test was used to compare sex distributions. We conducted TBSS analysis on each parameter map with the use of FSL. A nonparametric model was established, incorporating age, sex, and years of education as covariates. Nonparametric permutation testing with 5,000 permutations was conducted using the randomize command. The results were adjusted for multiple comparisons using threshold-free cluster enhancement (TFCE) correction. Statistical significance was set at *p* < 0.01. The final results were superimposed on the Johns Hopkins University (JHU)-ICBM-labels template to determine the anatomical locations in MNI space. The parameter values of the fiber bundles that showed statistically significant differences after TFCE correction were analyzed for correlation with MMSE and MoCA scores. Statistical significance was determined for correlations with a *p*-value less than 0.05, and Bonferroni correction was applied for multiple comparisons. Pearson correlation analysis was employed for normally distributed data, while Spearman correlation analysis was utilized for non-normally distributed data to calculate the correlation coefficient (*r*).

## 3 Results

We enrolled a total of 28 patients diagnosed with CAD and 30 HC in our study. The mean age of the CAD patients was 58.04 ± 11.86 years, while that of the HC was 53.90 ± 15.61 years. Among the CAD patients, 17 (60.71%) were male. Additionally, 16 individuals had a prior medical history of hypertension, 10 had a history of diabetes, 15 had a history of hyperlipidemia, 9 had a history of smoking, and 12 had a history of drinking. Detailed demographic and clinical information of the subjects can be found in Table 1. Compared with the HC group, CAD patients exhibited significantly lower scores on the MMSE (*p* < 0.01) and the MoCA (*p* < 0.001) (Table 1).

**Table 1:** Demographics and clinical data.

Variable	CAD (*n* = 28)	HC (*n* = 30)	*p*-value
Demographic information			
Male sex, No. (%)	17 (60.71%)	18 (60.0%)	0.224
Age (years), mean (SD)	58.04 ± 11.87	53.90 ± 15.62	0.913
Hypertension, No.	16	17	0.971
Diabetes mellitus, No.	10	9	0.643
Dyslipidemia, No.	15	11	0.196
Smokers, No.	9	13	0.380
Drinkers, No	12	14	0.771
Education, years	11.25 ± 4.94	12.50 ± 4.02	0.618
MMSE score	24.82 ± 2.78	28.07 ± 1.57	<0.01
MoCA score	22.89 ± 3.95	27.30 ± 1.15	<0.001

MMSE, mini-mental state examination; MoCA, Montreal cognitive assessment.

### 3.1 Comparison results between groups in terms of KFA, MK, AK, RK, FA, MD, and RD values

**Figure 1:**
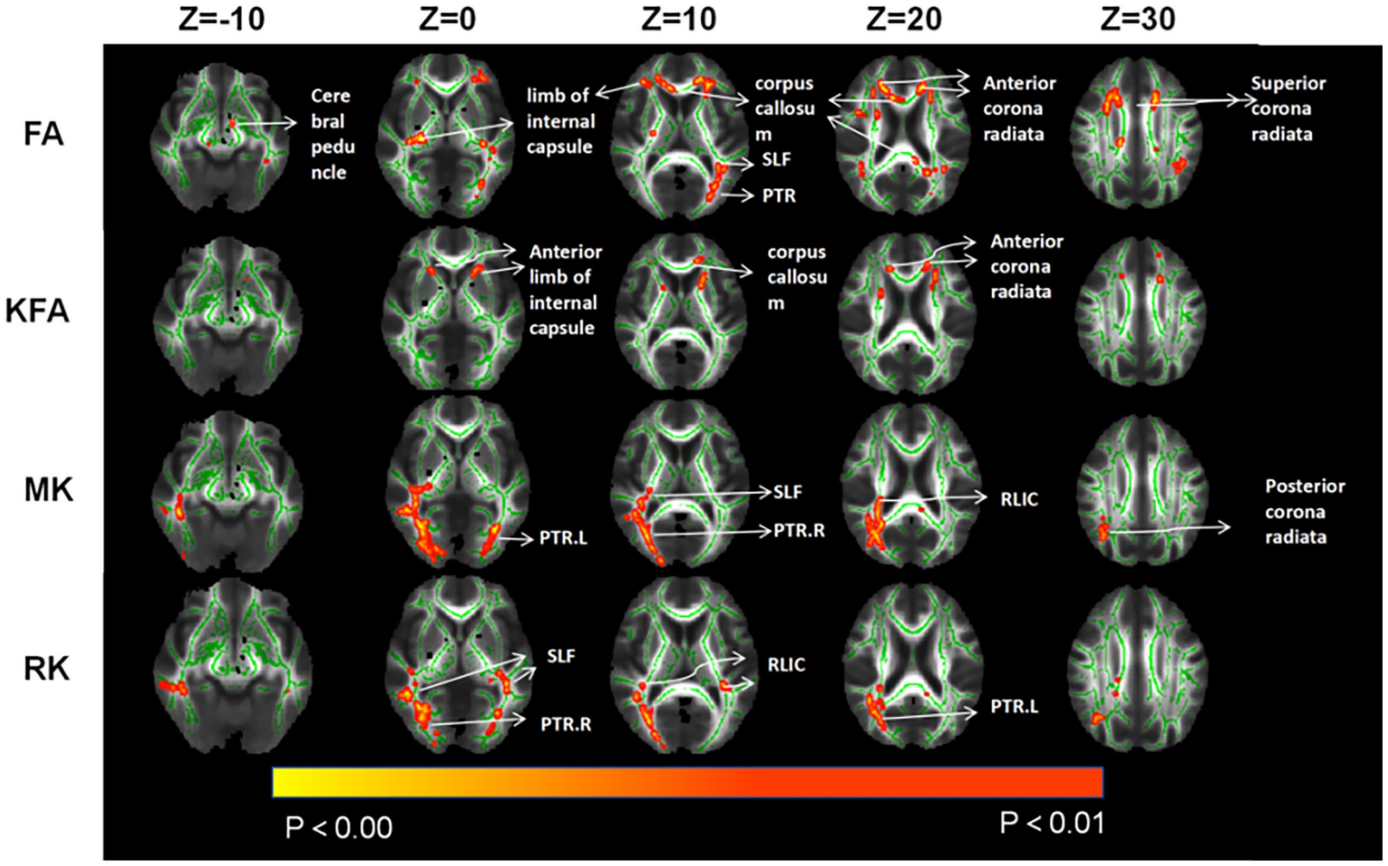
Compared to HC, TBSS analysis revealed significant differences in parameter values associated with changes in brain white matter fiber tracts for CAD patients. SLF, Superior longitudinal fasciculus; PTR, posterior thalamic radiation; RLIC, retrolenticular part of internal capsule.

**Figure 2:**
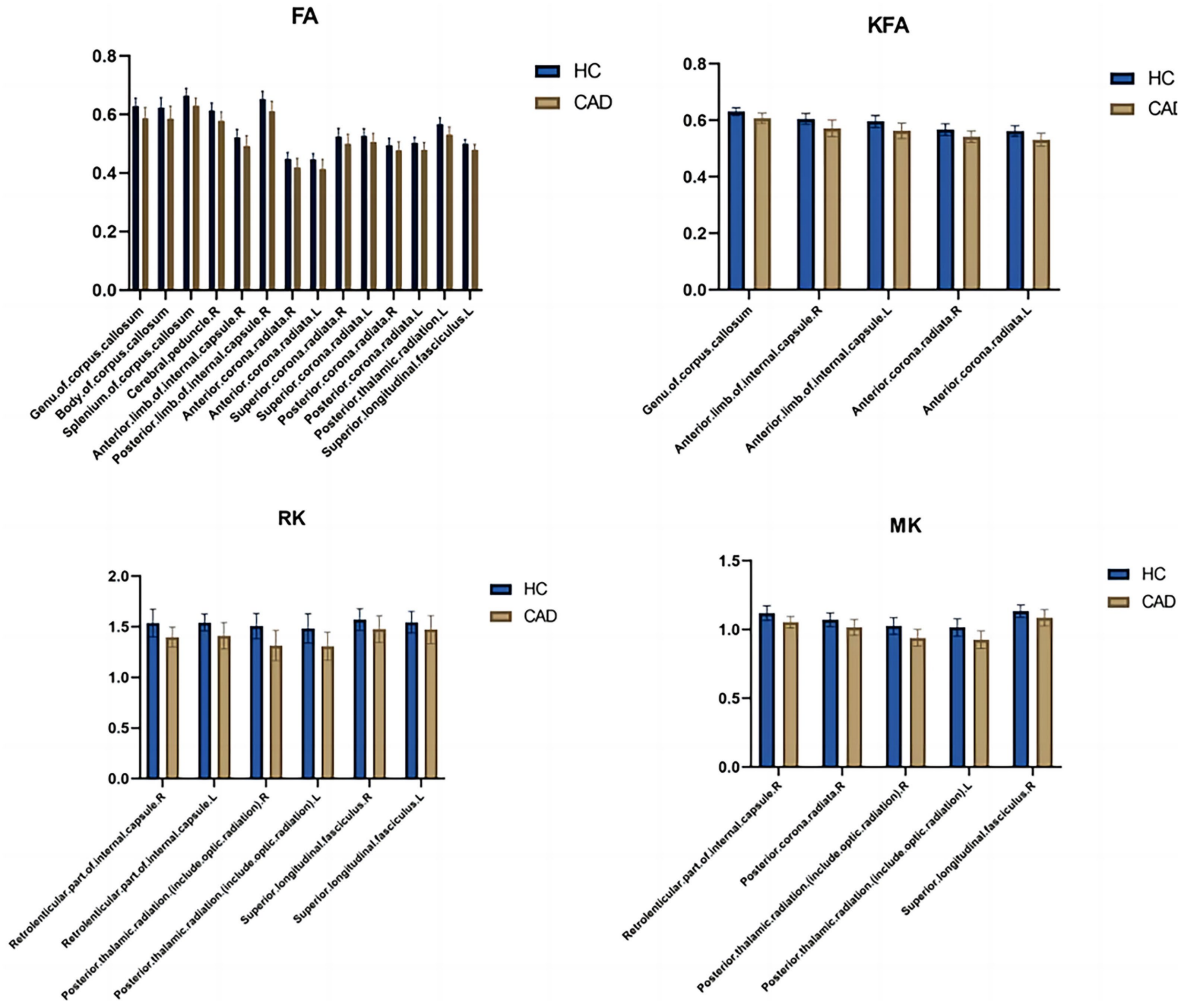
Bar graphs comparing the parameter differences of various white matterfiber bundles between the healthy control group and the CAD group. L, Left; R, Right; HC, Healthy Controls; CAD, Coronary artery disease.

In the CAD group, a significant decrease in FA values was observed in various brain regions. These regions included the superior corona radiata and corpus callosum (genu, body, splenium) bilaterally, the anterior corona radiata bilaterally, the posterior corona radiata bilaterally, the right posterior limb of the internal capsule, the right anterior limb of the internal capsule, the left posterior thalamic radiation, the left superior longitudinal fasciculus, and the right cerebral peduncle (*p* < 0.01, TFCE corrected) (Figure 1 and Table 2). Additionally, significantly decreased KFA values were found bilaterally in the anterior corona radiata, bilaterally in the anterior limb of the internal capsule, and in the genu of the corpus callosum in the CAD group (p < 0.01, TFCE corrected) (Figure 1 and Table 3). In the CAD group, we also observed significant decreases in MK values in specific brain regions. These regions included the right posterior corona radiata, the right retrolenticular part of the internal capsule, the posterior thalamic radiation (including optic radiation) bilaterally, and the right superior longitudinal fasciculus (*p* < 0.01, TFCE corrected) (Figure 1 and Table 4). In summary, there were significant differences in FA, KFA, MK, and RK values between the two groups in the aforementioned brain regions (Figure 2). Additionally, we found significant decreases in RK values bilaterally in the retrolenticular part of the internal capsule, bilaterally in the posterior thalamic radiation (including optic radiation), and bilaterally in the superior longitudinal fasciculus in the CAD group (*p* < 0.01, TFCE corrected) (refer to Figure 1 and Table 5). However, there were no statistically significant differences in MD and RD values between the CAD and HC groups (*p* > 0.01, TFCE corrected).

**Table 2:** The FA values of white matter fiber tracts in the CAD group were lower compared to the control group.

**White matter fiber tracts**	**Mean**	**SD**	**MNI**			***p***	***X***	***Y***	***Z***
Superior corona radiata R	0.50	0.03	19	-12	40	0.001
Superior corona radiata L	0.51	0.03	−19	−12	40	0.001
Anterior corona radiata L	0.41	0.03	−17	21	27	0.001
Anterior corona radiata R	0.45	0.02	17	21	27	0.001
Posterior corona radiata L	0.48	0.02	−20	−33	37	0.002
Posterior corona radiata R	0.48	0.03	20	−33	37	0.002
Genu of corpus callosum	0.59	0.04	−7	24	14	0.002
Body of corpus callosum	0.58	0.04	−15	16	27	0.002
Splenium of corpus callosum	0.63	0.03	20	−40	27	0.002
Posterior thalamic radiation L	0.53	0.03	−32	−41	14	0.002
Posterior limb of internal capsule R	0.61	0.03	27	−17	18	0.002
Anterior limb of internal capsule R	0.52	0.03	22	19	7	0.002
Superior longitudinal fasciculus L	0.48	0.02	−30	−23	−2	0.002
Cerebral peduncle R	0.58	0.03	13	−25	−15	0.002

MNI, Montreal Neurological Institute; L, left; R, right; SD, standard deviation.

**Table 3:** The KFA values of white matter fiber tracts in the CAD group were lower compared to the control group.

White matter fiber tracts	Mean	SD	MNI			*p*
			X	Y	Z	
Anterior corona radiata L	0.53	0.02	−23	27	13	0.001
Anterior corona radiata R	0.54	0.02	20	21	26	0.001
Anterior limb of internal capsule L	0.57	0.03	−20	9	14	0.001
Anterior limb of internal capsule R	0.56	0.03	23	7	14	0.001
Genu of corpus callosum	0.61	0.02	15	26	18	0.002

MNI, Montreal Neurological Institute; L, left; R, right; SD, standard deviation.

**Table 4:** The MK values of white matter fiber tracts in the CAD group were lower compared to the control group.

White matter fiber tracts	Mean	SD	MNI			*p*
			X	Y	Z	
Posterior corona radiata R	1.01	0.06	27	−40	23	0.01
Retrolenticular part of internal capsule R	1.05	0.04	34	−26	1	0.01
Posterior thalamic radiation (include optic radiation) R	0.94	0.06	29	−65	17	0.01
Superior longitudinal fasciculus R	1.08	0.06	43	−46	7	0.01
Posterior thalamic radiation (include optic radiation) L	0.93	0.06	−33	−60	1	0.01

MNI, Montreal Neurological Institute; L, left; R, right; SD, standard deviation.

**Table 5:** The RK values of white matter fiber tracts in the CAD group were lower compared to the control group.

White matter fiber tracts	Mean	SD	MNI			*p*
			X	Y	Z	
Retrolenticular part of internal capsule L	1.41	0.13	27	−40	23	0.01
Retrolenticular part of internal capsule R	1.40	0.10	39	−32	−2	0.01
Posterior thalamic radiation (include optic radiation) R	1.31	0.15	30	−67	1	0.01
Superior longitudinal fasciculus R	1.48	0.13	42	−44	8	0.01
Superior longitudinal fasciculus L	1.47	0.14	−42	−46	5	0.01
Posterior thalamic radiation (include optic radiation) L	1.30	0.14	−32	−60	2	0.01

MNI, Montreal Neurological Institute; L, left; R, right; SD, standard deviation.

### 3.2 Correlation analysis

**Figure 3:**
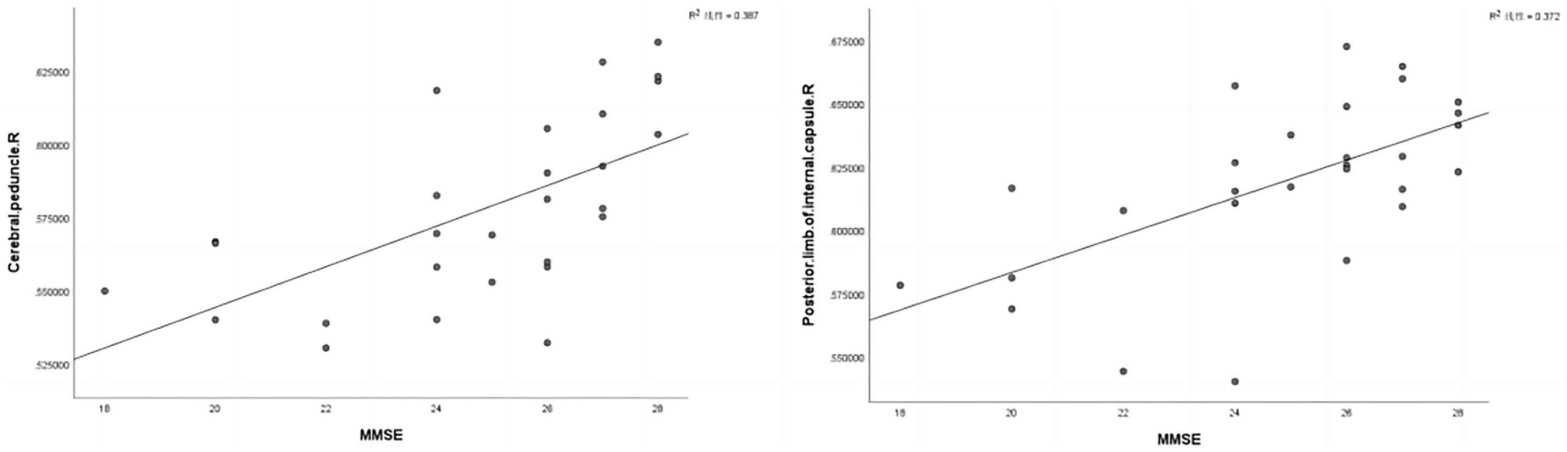
Correlation analysis was conducted between statistically significant fiber tract parameter values in the CAD group and MMSE scores. R, Right; MMSE, Mini-Mental State Examination.

In the FA map, the FA values of the right posterior limb of the internal capsule (*r* = 0.610, *p* = 0.001) and the cerebral peduncle (*r* = 0.622, *p* < 0.001) were positively correlated with MMSE scores (Figure 3 and Table 6). However, in the HC group, the fiber bundle parameters that showed statistically significant differences did not correlate with MMSE or MoCA scores (*p* > 0.05).

**Table 6:** The quantitative correlation between statistically significant fiber tract parameter values in the CAD group and MMSE and MoCA scores.

	Mean	SD	*r*	*p*
Cerebral peduncle R	0.58	0.03	0.629	<0.001
MMSE	24.82	2.78		
Posterior limb of internal capsule R	0.61	0.03	0.573	0.001
MMSE	24.82	2.78		

R, right; SD, standard deviation; MMSE, mini-mental state examination.

### 3.3 Pearson correlations between diffusivity and kurtosis parameters

**Figure 4:**
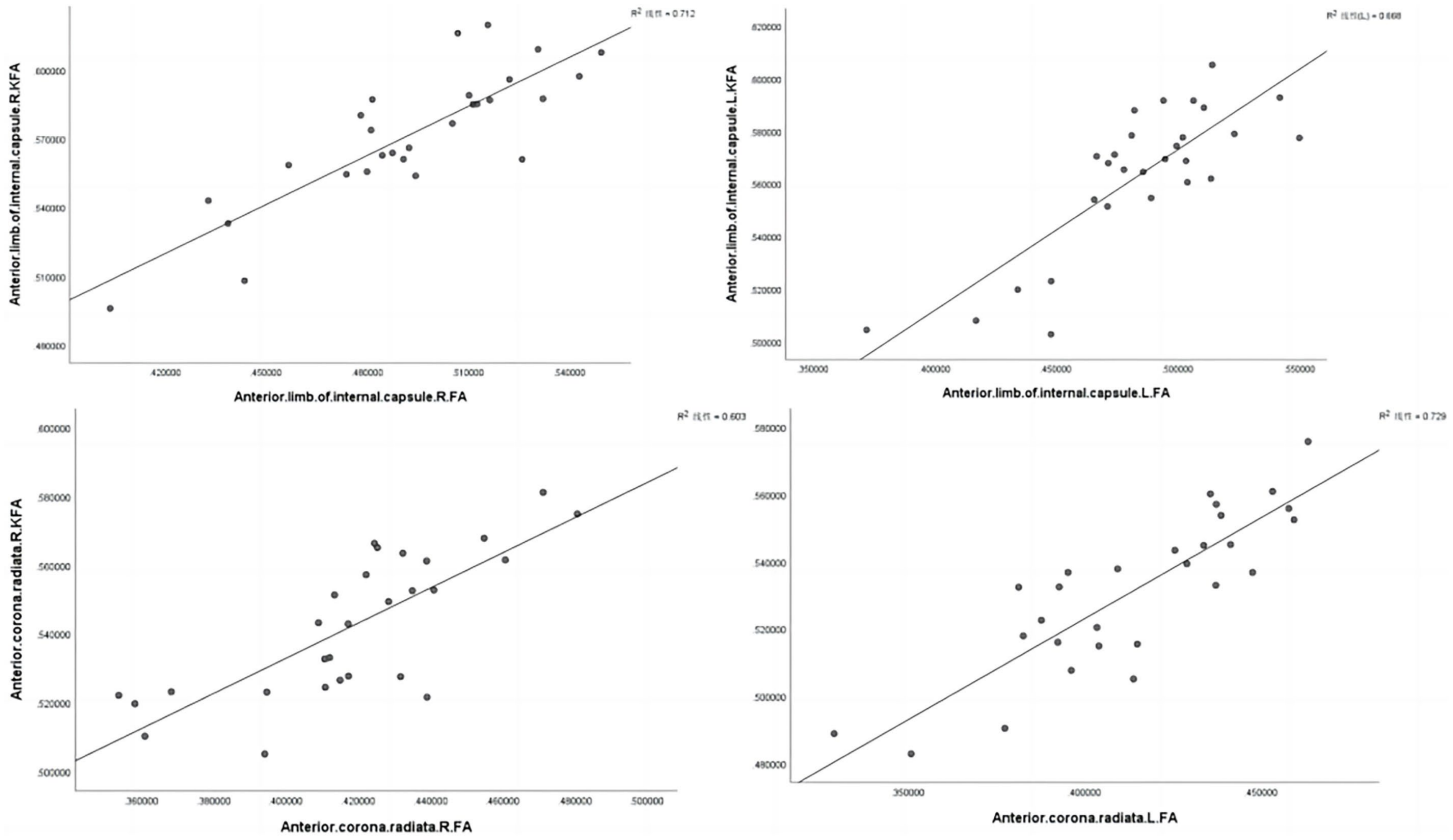
Correlations between diffusivity and kurtosis parameters from all ROIs. L, Left; R, Right.

We found significant positive correlations between the FA and KFA values in CAD patients, specifically in the left anterior limb of the internal capsule (*r* = 0.843, *p* < 0.001), right anterior limb of the internal capsule (*r* = 0.817, *p* < 0.001), right anterior corona radiata (*r* = 0.776, *p* < 0.001), and left anterior corona radiata (*r* = 0.853, *p* < 0.001) (Figure 4 and Table 7).

**Table 7:** The quantitative correlation between FA and KFA from all ROIs.

	Mean	SD	*r*	*p*
Anterior limb of internal capsule R	0.52	0.03	0.843	<0.001
Anterior limb of internal capsule L	0.53	0.03	0.817	<0.001
Anterior corona radiata R	0.45	0.02	0.776	<0.001
Anterior corona radiata L	0.41	0.03	0.854	<0.001

L, left; R, right; SD, standard deviation.

## 4 Discussion

According to research statistics (Virani et al., 2020), the prevalence of coronary artery disease (CAD) among adults over 60 is reported to be 14.9%. CAD is the leading cause of mortality and disability-adjusted life years (DALYs) globally (Ralapanawa and Sivakanesan, 2021). Lifestyle changes and improved living standards have contributed to the increasing incidence of CAD (Tsao et al., 2023). It is well established that cardiovascular complications can accelerate the progression of cardiovascular diseases, highlighting the need for increased attention to cardiovascular diseases and their associated complications (Lovell et al., 2019). Among these complications, CI is prevalent among CAD patients, with research confirming CAD as an independent international risk factor for CI (Balbaid et al., 2020).

DKI is a noninvasive diffusion imaging technique based on a non-Gaussian distribution mathematical model of the fourth-order three-dimensional completely symmetric tensor. Compared to traditional DTI techniques based on second-order tensor models, DKI is less affected by factors at multiple neural fiber crossings, resulting in higher measurement accuracy. It can more accurately reflect the isotropic movement of water molecules within tissues at the microstructural level, making it more suitable for describing the diffusion characteristics of white matter fiber bundles (Glenn et al., 2015). To the best of our knowledge, this is the first study to investigate alterations in white matter fibers in CAD patients using DKI parameters and the TBSS technique. Our study primarily observed that compared to HCs, patients with CAD exhibited decreased FA values in white matter tracts, including the corona radiata, corpus callosum, posterior thalamic radiation, posterior limb of internal capsule, and anterior limb of internal capsule. Additionally, decreased KFA values were observed in the corona radiata, anterior limb of the internal capsule, and genu of the corpus callosum. Furthermore, reduced MK and RK values were found in the retrolenticular part of the internal capsule, posterior thalamic radiation, and superior longitudinal fasciculus.

The corpus callosum is one of the major fiber bundles in the brain that connects the left and right hemispheres. It plays a crucial role in cognitive function. Specifically, the corpus callosum facilitates the development and maintenance of cognitive abilities by transmitting information and coordinating communication between brain regions on both sides. By carrying the neural fibers between the left and right hemispheres, the corpus callosum enables the exchange and coordination of information through electrical and chemical signals. This interhemispheric connection is essential for attention, memory, language, spatial perception, and emotional regulation. Therefore, the normal structure and function of the corpus callosum’s white matter fibers are vital for proper cognitive functioning in the brain. The body of the corpus callosum connects the posterior part of the bilateral frontal lobes and the entire parietal lobes, while the genu of the corpus callosum connects the anterior portion of the bilateral frontal lobes (Douaud et al., 2007; Salo et al., 2009). In our study, we observed a significant reduction in FA values in the white matter fiber bundles of the corpus callosum and a prominent decline in the KFA values in the genu region of the corpus callosum in patients with CAD when compared to HCs. The FA value reflects the directional properties of water molecules in a magnetic field and is associated with the integrity of myelinated fibers and the density of white matter nerve fibers. When there are changes in the microstructure of myelin or disruption of myelin integrity, the FA value decreases. A decrease in KFA values of white matter fiber bundles in DKI indicates a reduction in complexity and weakening of nonlinear features. Our research findings indicate that the integrity of the corpus callosum white matter fibers is impaired in CAD patients, which may be closely associated with the decline in cognitive function observed in CAD patients. Studies by Mielke et al. have shown that the loss of corpus callosum white matter fibers leads to widespread disconnection in cortical and subcortical networks, resulting in CI (Mielke et al., 2009). Raghavan et al. (2020) suggested that the decrease in corpus callosum FA values could serve as a biomarker for cerebrovascular diseases and as a predictive factor for cognitive impairment in MCI patients. For KFA, Falangola et al. found that the KFA values of the corpus callosum in 3xTg-AD mice were significantly lower than those in the control group. Additionally, the morphological quantitative values of myelin basic protein immunoreactivity were significantly decreased in the 3xTg-AD mice. Based on these findings, they hypothesized that demyelination could lead to a decrease in the compactness of the white matter in the brains of 3xTg-AD mice, which is manifested by a significant decrease in KFA values (Falangola et al., 2013). For our study, the decreased KFA values in CAD patients suggest a reduced compactness of the corpus callosum white matter microstructure, which may be closely associated with the decline in cognitive function.

The corona radiata is primarily responsible for connecting the cortical and subcortical regions, playing a significant role in cognitive functions related to memory and language processing. These fiber bundles are composed of axons that extend from the cerebral cortex to different subcortical structures, enabling the exchange of information between distinct brain areas (Olivo et al., 2017). Our research findings revealed that, compared to HCs, patients with coronary artery disease (CAD) exhibited a significant decrease in FA values in the upper, anterior, and posterior regions of the bilateral corona radiata. Additionally, KFA values significantly decreased in the anterior regions of the bilateral corona radiata, while MK values significantly decreased in the posterior region of the right corona radiata. These findings are consistent with previous studies on cognitive impairment. A study conducted by Falangola et al. explored MCI patients using DKI sequences. The findings of their study indicate that, compared to HC, MCI patients exhibit white matter fiber bundle loss in the corona radiata, resulting in a significant decrease in the complexity of the brain parallel to the direction of the fiber bundles (Falangola et al., 2013). This study discovered alterations in the microstructure of the corpus callosum and corona radiata in patients with CAD. Previous research has similarly found white matter fiber structural damage in the corpus callosum and corona radiata among individuals with MCI. Therefore, we hypothesize that the cognitive decline observed in CAD patients may be related to a decrease in the density and complexity of brain white matter microstructures, including the corpus callosum and corona radiata.

The internal capsule, cerebral peduncle, and posterior thalamic radiation are classified as projection fibers. The literature has reported that these regions are less affected in CI. However, as the understanding of MCI deepens, an increasing number of studies indicate more widespread damage to projection fibers in MCI (Pettersson et al., 2005). Our study revealed significant decreases in FA values in the anterior–posterior limbs of the right internal capsule, the posterior limb of the left thalamus, and the cerebral peduncle in CAD patients compared to HCs. Additionally, bilateral decreases in KFA values were observed in the anterior limbs of the internal capsule, along with reductions in MK and RK values in the internal capsule and bilateral posterior limbs of the thalamus. The decline in MK suggests a decrease in tissue complexity and integrity, while the decrease in RK may reflect reduced radial diffusion within the tissue. These alterations in DKI metrics of white matter tracts indicate white matter fiber damage, which may be closely related to cognitive impairment in patients with coronary artery disease. These findings are consistent with previous research results (Chen et al., 2023). The results of our study revealed a positive correlation between the FA values of the right posterior limb of the internal capsule and the cerebral peduncle were positively correlated with MMSE scores. This suggests a close relationship between FA values in the right posterior limb of the internal capsule and the crus of the right cerebrum and clinical cognitive performance. At present, we have not found any articles specifically discussing the relationship between DKI and CI in CAD patients. However, a study on a population with pure cognitive impairment found that DKI metrics were closely related to cognitive function scores in regions such as the posterior limb of the internal capsule (Allen et al., 2019). This finding is consistent with our research results. The posterior limb of the internal capsule contains fiber tracts that are involved in language and cognitive functions, contributing to the execution of cognitive functions. Moreover, white matter fiber tracts in the brain, including those in the internal capsule, play crucial roles not only as conduits for information transmission but also as bridges for coordination and communication between different brain regions. They are also involved in neural regulation processes. This suggests that damage to the posterior limb of the internal capsule and the white matter fiber tracts in the brain, which may occur in patients with CAD, can impede information processing and communication, thus closely correlating with the development of cognitive impairment.

The superior longitudinal fasciculus is a bundle of neural fibers that serves to connect various brain lobes within the same hemisphere and is involved in the transmission of multiple sensory information (Geldmacher et al., 2007). Previous literature has indicated that impairments in the transmission of information related to the superior longitudinal fasciculus in the frontal lobe can lead to visual disturbances in patients with CI. Meng et al. (2012) discovered a significant decrease in bilateral superior longitudinal fasciculus FA values in patients with MCI, which also showed a positive correlation with MMSE scores. In this study, CAD patients with CI showed a decrease in FA, MK, and RK values of the superior longitudinal fasciculus, consistent with previous reports on CI. These findings suggest a reduced integrity of the superior longitudinal fasciculus in CAD patients, leading to a decrease in the strength of connections between the cerebral hemispheres on the corresponding side and subsequently resulting in a decline in cognitive function. We also observed a significant correlation between kurtosis and diffusion coefficient parameters (FA and KFA), which is consistent with the study of NJ G. The findings indicated that alterations in diffusivity were accompanied by changes in diffusional non-Gaussianity.

## 5 Limitation

Our study does have certain limitations that should be acknowledged. One of the main limitations is the relatively small sample size, consisting of only 28 patients with CAD and 30 HC participants. This small sample size may limit the generalizability of our findings and reduce the statistical power of our analyses. It is important to note that our study subjects were recruited from a single medical institution, which may introduce recruiting bias and may not fully represent all populations with CAD. Therefore, further studies with larger and more diverse samples are needed to validate our results. Additionally, in terms of neuropsychological examination, we only utilized the MMSE and MoCA scales to assess the cognitive function of the subjects. While these scales provide valuable insights, they may not comprehensively evaluate all aspects of cognitive decline. In future studies, it will be important to include a broader range of cognitive assessment tools to obtain a more precise understanding of the degree of decline in various cognitive functions. Furthermore, our study focused solely on the differences in white matter fiber tracts between CAD patients and HCs, without considering other potential factors that could influence the research results. Factors such as drug treatments and individual differences among patients may contribute to variations in cognitive function. Future studies should aim to incorporate these factors into the research design to provide a more comprehensive analysis.

## 6 Conclusion

Patients with CAD exhibit a decrease in the tightness and complexity of brain white matter microstructure. The TBSS-based DKI analysis of whole-brain white matter is capable of objectively reflecting the characteristic white matter damage in CAD patients. Furthermore, certain DKI metrics are significantly correlated with cognitive function, providing evidence for the auxiliary diagnosis of CAD patients with CI.

## Data availability statement

The datasets presented in this study can be found in online repositories. The names of the repository/repositories and accession number(s) can be found at: https://doi.org/10.6084/m9.figshare.23703582.

## Ethics statement

The studies involving humans were approved by Ethics Committee of Shandong Provincial Hospital. The studies were conducted in accordance with the local legislation and institutional requirements. The participants provided their written informed consent to participate in this study.

## Author contributions

TL: Funding acquisition, Software, Writing – review & editing. RQ: Methodology, Software, Writing – original draft. CL: Project administration, Software, Writing – review & editing. LL: Data curation, Resources, Writing – review & editing. XW: Conceptualization, Data curation, Methodology, Resources, Software, Writing – review & editing. LW: Conceptualization, Data curation, Methodology, Project administration, Software, Supervision, Writing – review & editing.

## Footnotes

^1^ https://www.nitrc.org/projects/dke
